# Supplementary material for: Complexome profiling of the Chlamydomonas psb28 mutant reveals TEF5 as an early PSII assembly factor
Source: Plant Cell. 2025 Mar 17;37(6):koaf055. doi: 10.1093/plcell/koaf055 (PMC12164590; doi:10.1093/plcell/koaf055)
Supplement: koaf055_Supplementary_Data [file koaf055_supplementary_data.zip › psb28_interactive.zip!/plots/intensity/qualityPlots/peptides/_Cre02.g114450.t1.2.html]

### Cre02.g114450.t1.2

Trivial name:   
SeqCoverage [median]: 14.40  
Mass: 18.96 kDa  
  
 

| Sequence | MissedCleavage | Mass [Da] | wtIntensity | muIntensity | Score |
| --- | --- | --- | --- | --- | --- |
| LAQADEAFEK | 0 | 1120.54 | 437.43 | 1108.71 | 126.31 |
| LAQADEAFEKSDTLK | 1 | 1664.83 | 663.40 | 2602.07 | 63.04 |
| SLSELISGGGK | 0 | 1046.56 | 1524.59 | 3779.68 | 108.98 |
|  |  |  | 2625.4 | 7490.5 |  |
